# Can We Tune Our Pediatric Patients?

**DOI:** 10.5005/jp-journals-10005-1107

**Published:** 2011-04-15

**Authors:** Ritu Jindal, Rajwinder Kaur

**Affiliations:** 1 Professor and Head, Department of Pedodontics, National Dental College and Hospital, Dera Bassi, Punjab, India; 2 Postgraduate Student, Department of Pedodontics, National Dental College and Hospital, Dera Bassi, Punjab, India

**Keywords:** Dental anxiety, Music.

## Abstract

For the pedodontic team, a child’s dental anxiety poses major management problems. Previously, wide variety of aversive techniques have been used with varying success rates to manage anxious child patients. The present trend advocates the use of nonaversive techniques like distraction in the management of anxious pediatric patients. So the aim of this study is to compare the effect of audio distraction with the normal set up operatory. Thirty patients of age between 4 and 8 years were included in the study. Each patient had gone through four dental visits. Anxiety was measured using Venham’s picture test. The values obtained were tabulated and statistical analysis and concluded that audio distraction did decrease the level of anxiety in anxious pediatric dental patients to a significant level during the restorative procedure visit (3rd) and invasive procedure visit (4th).

## INTRODUCTION

For many years, fear and anxiety associated with dental treatment are well-recognized factors and have a negative impact on patient’s willingness to get dental treatment.^1^ Dental fear and anxiety (DFA), a common occurrence characterized by an essential and inevitable emotion that appears as a response to various dental procedures. It varies in intensity from one patient to another, ranging from a simple nervousness to dental anxiety, which is a sentiment of fear often unjustified and can disappear spontaneously or amplify, thus defining dental phobia.^2^

The issue of dental fear and anxiety has been studied extensively and presents a significant problem to patients and dentists alike. A sizeable proportion of the population are anxious about dental treatment, and it is recognized that this can act as a barrier to oral health.^3^

Etiological factors for anxiety in child patient are classified into two types.^4^

- Exogenous factors/dental factors:
  - Direct experience
  - Indirect experience or familial trait
  - Fear of unknown.
- Endogenous factor/nondental factor (psychological factor):
  - Trait anxiety of children
  - General behavior problem of children
  - Temperament of child
  - Socioeconomic status.

Managing the anxiety of a child patient so as to become cooperative patient is critical to the success of dental treatment. Although, traditional techniques may be successful but the attitude of parents and dental professionals toward these techniques is changing. For example, immobilization in a papoose board, although effective, has been shown to be unacceptable among a majority of patients.^5,6^ Regarding the pharmacological method of management parents hesitate because of the medical risk.

So present trend advocates the use of nonaversive behavior management techniques which may be equally effective and more acceptable to parents, patients and practitioners.

Therefore, the aim of this study is to compare the effect of audio distraction with normal set-up operatory in the management of anxious pediatric dental patients.

## MATERIALS AND METHODS

Thirty patients of age between 4 and 8 years with first dental visit, who were well-oriented with time and place, were included in the study.

Children with lack of orientation and mental and physical disabilities were excluded from the study.

Before beginning with the study consent was taken from the patient’s parents along with brief dental and medical history of patient.

Children were divided into two groups with 15 patients in each group.

**Fig. 1:**
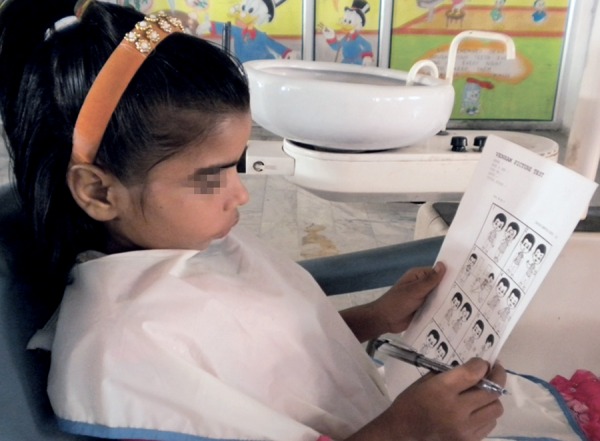
Patient under control group

**Fig. 2:**
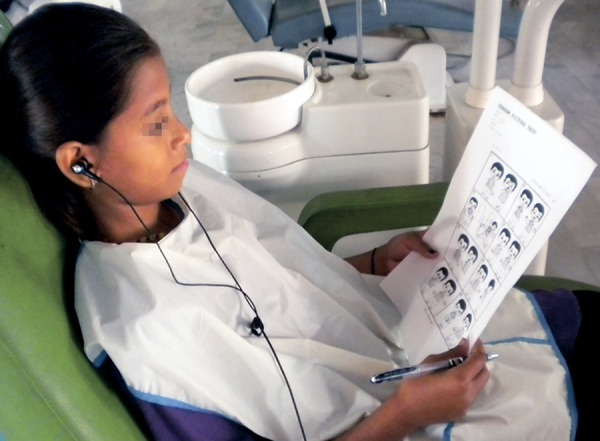
Patient under music group

- *First group:* Control group―In the control group, treatment was done under normal set-up operatory (Fig. 1)
- *Second group:* Music group―In the music group, each child listened to audio presentation through headphones throughout the course of the treatment. The choice of the type of music was according to the patient’s selection (Fig. 2).

Each child had four dental visits as follows:

1. Screening visit
2. Oral prophylaxis visit
3. Restorative procedures without the need of a local anesthetic injection
4. Invasive procedures necessitating the need of a local anesthetic injection.

**Fig. 3:**
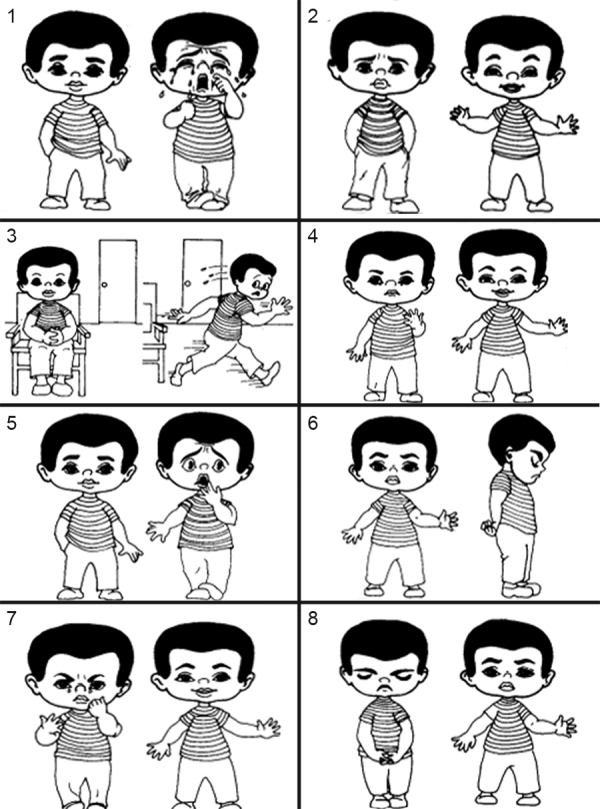
Venham’s picture test

During the course of treatment, child’s anxiety level was assessed using Venham’s picture test (Fig. 3).

The picture test is a projective self-report measure of anxiety. It consists of eight pairs of cartoons of a child exhibiting various emotional states: Surprise, fear, anger, etc. The child was instructed to choose the picture that best reflects how he/she feels. The more emotional figure of the pair of cartoons is scored one, whereas the more emotionally neutral figure is scored zero. Therefore, the scale has a range of zero to eight. It is quick to administer 2 to 3 minutes.^7^

The values obtained were tabulated and subjected to statistical analysis.

## RESULTS

Self-reported measure of anxiety: Venham’s scale was administered two times to each patient: Prior to each treatment session and immediately following the treatment on subsequent visits.

Anova (an analysis of variance) and Mann-Whitney test were performed to find out the mean of pre- and posttreat-ment values (Tables 1 and 2).

The results showed that the mean sum of scores for the examination visit was similar in all the patients both in the control (6.00) and music (6.00) groups. On second visit, during prophylaxis mean sum of scores in the control (6.50) group and music (6.25) group were even almost same, which was statistically insignificant. During the restorative visit, mean sum of scores for control group and music group was 6.57 and 5.56 respectively and during 4th visit mean sum of scores in control group and music group was 5.07 and 4.38 respectively, which were statistically significant with a p < 0.05.

**Table 1:** **Table 1:** Means

*Groups* *visit*				*1st visit*		*2nd visit*		*3rd visit*		*4th*	
Control		Mean		6.00		6.50		6.57		5.07	
group		N		15		15		15		15	
		Std. deviation		0.000		1.092		1.222		0.730	
		Minimum		6		5		4		4	
		Maximum		6		8		8		6	
		Median		6.00		6.00		6.50		5.00	
Music		Mean		6.00		6.25		5.56		4.38	
group		N		15		15		15		15	
		Std. deviation		0.000		0.577		0.727		0.885	
		Minimum		6		5		4		3	
		Maximum		6		7		7		6	
		Median		6.00		6.00		6.00		4.00	
Total		Mean		6.00		6.37		6.03		4.70	
		N		30		30		30		30	
		Std. deviation		0.000		0.850		1.098		0.877	
		Minimum		6		5		4		3	
		Maximum		6		8		8		6	
		Median		6.00		6.00		6.00		5.00	

**Table 2:** **Table 2:** Mann-Whitney test

*Groups*				*N*		*Mean* *rank*		*Sum of* *ranks*	
1st visit		Control group		15		15.50		217.00	
		Music group		15		15.50		248.00	
		Total		30					
2nd visit		Control group		15		16.11		225.50	
		Music group		15		14.97		239.50	
		Total		30					
3rd visit		Control group		15		19.64		275.00	
		Music group		15		11.88		190.00	
		Total		30					
4th visit		Control group		15		19.07		267.00	
		Music group		15		12.38		198.00	
		Total		30					

The test of significance showed that results were significant in the third and fourth visits with a p < 0.0051 (Table 3).

**Table 3:** **Table 3:** Test of significance

		*1st visit*		*2nd visit*		*3rd visit*		*4th visit*	
p-value		1.000		0.694		0.011*		0.028*	

*Signify p < 0.005

## DISCUSSION

Anxiety in patients still poses a significant problem for the practice of dentistry, so detecting and assessing dental anxiety among child patients with some valid method of measurement is necessary. Therefore, aim of the present study was to evaluate the efficacy of audio distraction in the management of anxious pediatric dental patients by using Venham’s picture test during four different treatment procedures.

Venham’s picture test, which was used in this study, is one of the reliable measures of self-portrayed anxiety in children. It is very effective in measuring the emotional state of the child at the chairside (Venham et al, 1977).^8,9^

As per the observations made in the present study, maximum level of anxiety was apparent during the restorative procedure visit and invasive procedure visit. But in the music group, the anxiety level was shown to be reduced, confirming the physiologic relaxation due to music distraction.

The choice of music was left to the patients because playing familiar music which might have helped the child gain control over the unpleasant stimulus and give them a feeling of being in familiar environment as done in a previous study.^10^

Reduction in anxiety can be attributed to two reasons as supported by two others.^11^

- A child listening to music tends to close his/ her eyes to concentrate on the audio presentation, there by screening out the sight of dental treatment
- The sound of music will eliminate the unpleasant sounds in dental clinic like the sound of air rotor handpiece.

These above two advantages coupled with the effect of music will reduce the anxiety and provide relaxation and also help the dentist to effectively manage the anxious patient. We do confer that music did reduce the anxiety significantly. Music distraction may be helpful as an adjunct along with other behavior management techniques; therefore, further research in this field is necessary along with other nonaver-sive techniques.^12^

In contrast, our results contradict few studies that show no effect of music distraction.^13^

Regarding the Venham’s picture test, this has been proved to be a reliable measure of self-portrayed anxiety in children. Venham’s picture test has its own limitations such as the figures on the cards are all male; this may present problems if the young patient is a girl. In addition, some of the figures are vague in what they are depicting. Further studies with a larger sample size and modification of pictures to suit a girl patient are needed.^14^

## CONCLUSION

1. Audio distraction decreased the level of anxiety in anxious pediatric dental patients although to a significant level during the restorative procedure visit (3rd) and invasive procedure visit.
2. Despite lack of any relief from pain, the patient had an overwhelming positive response to the music presentation and looked forward to hear it in the subsequent visits.

At present, it is not possible to say whether the apparent reduction of anxiety would continue over a longer period , or whether it is partially produced by the good effect of ‘novelty’ where anything new and special can be expected to improve a patient’s reactions.

Perhaps it is time for further resurgence of interest in the whole subject of auditory stimulation in the reduction of anxiety in the dental surgery, for there are still many unanswered questions in this field.^15^

The results have to be interpreted with caution and will only be totally acceptable, if repeated trials support these preliminary findings.
